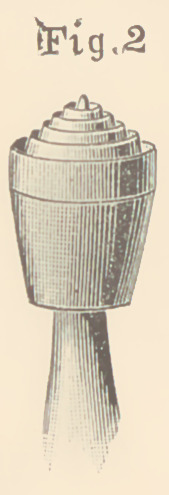# Current News and Opinion

**Published:** 1885-08

**Authors:** 


### Current News and Opinion.

### AMALGAM SOLVENT.

In the July number of the INDEPENDENT PRACTITIONER, page 393, occurs a statement by Dr. Teague, that "a solvent of at least some grades of amalgam does pervade the oral cavity."

I should like to have him examine the specimens that I enclose herewith, and see if he does not find all the conditions of which he speaks upon the underside of both fillings. I mean that portion next the tooth.

Certainly no solvent has been at work here, but plainly some one or more of the component parts of the mass failed to get its proportion of the solvent—that is, the mercury—and never became a solid.

I think we have all seen the converse of this also, where so much mercury was used that it became a solvent for portions of the filling that were already set. These re-dissolved portions, not becoming hard again, were easily rubbed out and washed out by food and saliva.

If Dr. Teague desires to experiment in this direction, let him take some amalgam that has just begun to set, and mix it up with some more mercury and note its condition afterward, and let us know if the conditions described by him are not explicable on other grounds than those he has in mind in his criticism.

The component parts of most amalgams unite with the mercury with varying degrees of intensity, and in some of the coarser grades I think it quite possible that the silver should take its quantum of mercury and become set before the tin, gold, platinum, copper, or other metals comprising the alloy, have even become softened. The usual mode of mixing in a mortar would cause unequal combination. Fletcher's mode of shaking the alloy with the mercury in a tube is far more accurate. E. A. BOGUE.

29 E. 20th Street, New York.

### ANSWER.

The specimens from Dr. Bogue are received. I have examined them as well as I can with apparatus at hand. My opinion is that in each case, when the amalgam was inserted, the cavity was not perfectly freed of chippings, as "the powder" mentioned I find to be particles of tooth-substance, gold foil and amalgam. It may be that barely enough mercury was given to the fillings, and in pressing the mass upon the debris in the cavity an irregular or rough surface underneath was made. It was therefore an easy thing for particles of the filling to become detached and mingle with "the powder" already there, especially if the filling became loose and "wabbled" in the cavity.

The cases I had in mind when I penned the piece for the INDEPENDENT PRACTITIONER, were a decided disintegration of the wearing (outside) surface. I took it to be from chemical action. Dr. Bogue's theory may be correct, yet the fillings seemed quite compact and strong. B. H. TEAGUE.

AIKEN, SOUTH CAROLINA.

---

### WILLIAMS ENGINE GOLD CONDENSER.

The cuts of this instrument were not finished in time for insertion in the article of Dr. Bodecker. As we wish to make the exposition of the system complete in every way, we insert the illustration here. Fig. 1 represents the instrument in its natural size. Fig. 2 shows it enlarged four times, to represent the details of its construction. The advantages claimed for it are, that it carries the gold forward and at the same time spins it out laterally ; that with it the gold can be picked up as with a pair of pliers ; that it answers a good purpose as a hand-plugger, and that it leaves the surface of the filling in the proper condition for the reception and easy condensation of the next cylinder or pellet.

Fig. 1

Fig. 2

---

### MRS. LUCRETIA L. BRACKETT.

Those who knew the peculiarly tender affection that existed between Dr. C. A. Brackett and his mother, and the devotion of each to the other, will sincerely sympathize with him in her death, which occurred at Newport, R. I., on the 11th ult. Hers was indeed one of those gentle and pure natures which are only known and fully appreciated by those who live in the closest relationship.

### GUTTA-PERCHA CAPPINGS.

Having seen nothing in the journals advising against the employment of gutta-percha as a capping for exposed pulps, while much has appeared urging its use both as a capping and as a non-conductor under metal fillings, I feel it a bounden duty to utter a note of warning. It is a well-known fact that gutta-percha possesses sufficient expansive qualities to make it a valuable agent in separating teeth. We have all observed this property, in a marked degree, when buccal cavities have been filled with it. No matter how carefully the work has been done, or how neatly and smoothly the material has been packed, a few months later we are sure to find it bulging from the cavity to such an extent as to make us feel as if we had used at least one-fourth too much material. This same change takes place when it is used over an exposed pulp, and confined under a filling. Sufficient pressure is exerted to produce inflammation and death of that organ.

When used as a non-conductor under large metallic fillings it will, in almost every case, expand to such a degree as to force the filling out, even if it has to fracture the walls of the tooth to do it. We can most heartily recommend it as a root-filling, but in such cases the roots should be filled with gutta-percha, and the pulp-chamber with gold, or some of the cements. THOMAS T. MOORE.

### INGENIOUS.

There are among dentists some minds of great originality. We know of one who packed his vulcanizer top with vulcanizable rubber. It stopped its leaking, but when the case came out it was taken out through a hole made in the bottom with a cold-chisel.

Another regularly oiled the thread of the top until, in his efforts to unscrew the greasy top, he had worn out the slots for the wrench. Finally, a more than usually liberal supply of oil afforded enough gum, when associated with the inability to make the wrench hold, to force him, too, to take a case out through the bottom.

### AMERICAN DENTAL ASSOCIATION.

The twenty-fifth annual session of the American Dental Association will be held at Minneapolis, Minnesota, commencing at 11 A. M., Tuesday, Aug. 4th, 1885. GEO. H. CUSHING,
Rec. Secretary.

### MIKE AND THE DOCTOR.

A dentist of Detroit, an officer of the American Dental Association, visiting in St. Louis not long since, was robbed of all his portable property—a pocket-book containing a few car tickets, and a newspaper clipping of a piece of poetry, entitled "Jim, our Jim" (Blaine). A few days since he received his pocket-book by express, with the following letter, which speaks for itself:

I always thort a dentist hadd a plentey of munney, but you are the porest kuss I ever saw. Just to think I run risk of gettin' collered and going to the pen and onely got this bloke of a car ticket boodel. are you travelling on your Cheke beatin ralerodes and hotels and hain't got no rocks or are you on a mishionary or a brace game lay i onely knowd bv aksident what your name is and where you live and what is your perfeshion and i am to dam proud to keep this babey i haint got no use for a jim blane man i mite of knowed he was a fluke a chump and i piped you of 2 days tried to fake your super but you was to fly never seen a pore denttist befor. i play pool you are a dam fraud i woulddint let you pull a toothe out of a rake. if them tickits was good for a drink i woulddint send them to you and i dont give my right name. you would blo the gaff. all the cops knowes me and I have done time.

MIKE.

---

### SENSITIVE DENTINE.

What I wish to show is that dryness is the best obtundent of pain in sensitive dentine, and that the efficiency of most of the agents now employed for this purpose is due to their power to bring about this result, and that the after-troubles experienced from thermal changes are due to the metallic filling having been packed into a damp bed, and that soft neural matter is in actual contact with, or in close proximity to, the metal plug without any protection; or if any application was made the parietal surface of the cavity was preoccupied with moisture, and not in a condition to take it up, instead of having a dry, hard surface of devitalized bone, saturated with some non-conducting material insoluble in water, capable of resisting any reasonable percussion from the mallet, and which shall form a natural cap over the pulp.—*Dr. R. F. H. King—Journal of the British Dental Association.*

---

### NATIONAL ASSOCIATION OF DENTAL EXAMINERS.

The next regular meeting of the National Association of Dental Examiners will be held at Minneapolis, on Tuesday, August 4, 1885. A full representation is very much desired.

GEO. H. CUSHING,

Secretary.

### EXTIRPATION OF THE LARYNX.

A very interesting and successful surgical operation was performed at the Buffalo General Hospital, June 28th, by Dr. Roswell Park, Professor of Surgery in the University of Buffalo, Medical Department, in the presence of members of the Faculty and the Hospital staff. The patient was a physician, 64 years of age, who was suffering from a malignant growth in the larynx. He had been under the care of several famous specialists in different cities. Not long before the death of Dr. Louis Elsberg, he removed a large amount of the growth by intra-laryngeal method. Dr. Park removed the cricoid and thyroid glands, with about two inches of the trachea. The hyoid bone and the uvula were taken out, and the œsophagus opened at the wound, that the man may be artificially fed, for, of course, with the removal of the hyoid bone all command of the muscles of the mouth and pharynx is lost. The delicacy of the operation, which lasted just an hour, may be understood when the character of the operation is comprehended. Dr. Park intends to insert an artificial larynx, containing reeds to serve in place of the removed vocal cords, and which he thinks will restore speech sufficiently to enable the patient to make himself understood. This is the third time that the operation has been performed in this country. At the time of writing this paragraph, nearly a month after the operation, the patient is doing well.

---

### A DENTURE IN THE STOMACH.

The *Deutsche Med. Zeit.* gives an account of a novel method for the removal of an artificial dental plate that had been accidentally swallowed. It proves that a little ingenuity is sometimes worth more than all the knowledge of the schools.

Dr. Geisselbrecht, a dentist of Furst, was sent for one night to attend a servant girl who had swallowed her artificial teeth, a plate which the *Zeitung*, with characteristic professional intelligence, says had upon it "four canines and two bicuspids." The denture could not be seen, but was felt in the larynx, and pushed through the cardiac orifice. Dr. G. reflecting that the gold clasps of the rubber plate and the many sharp points would prevent or retard its passage through the digestive canal, as well as probably injure the intestines, set his wits to work to smooth its journey. He cut spool cotton into small pieces, and incorporated them with the white of eggs, beaten up to a froth, and made the girl swallow a quantity of it. The result was eminently successful, the plate being passed in due time without pain or material inconvenience, and upon examination it was found completely invested with the thread, and with its sharp points well protected by their entanglement.

---

MAGITOT: CONCERNING THE SYPHILITIC ORIGIN OF RACHITIS.

The author's paper is an additional criticism in opposition to the theory of Parrot, that rachitis is merely an expression of congenital syphilis. The criticism is aimed at the "*syphilis dentaire*" of Parrot, *i. e.*, the deformed teeth, from the presence of furrows and crescentic notchings, which are considered by him (Parrot) as an indication of syphilis. Three questions are propounded by Magitot. The first is, is the erosion of the teeth a characteristic and unfailing sign of hereditary syphilis (as Parrot considers it)? The answer is, no, for (*a*) it is not a constant symptom; (*b*) those who possess it are known to have acquired chancre; (*c*) it occurs in cases in which no traces of syphilitic history is discoverable; (*d*) it sometimes occurs in dogs, cattle and other animals which are absolutely insusceptible to syphilis. The second question is, does hereditary syphilis have, as a necessary consequent, notable trophic disturbances of the dental apparatus? The answer is, yes, without doubt, but the phenomena which are presented are, usually, small, misshapen, conical teeth, defective anatomically and chemically, and late in their appearance. The third question is, what are the causes of the erosions in question, and the mechanism of their appearance? The answer is, a dystrophy of the two dental tissues, the enamel and the ivory, but not an atrophy, as Parrot asserts. The primary cause is to be found in certain disturbances of the nervous system and the general nutrition, and especially in conditions of a convulsive character, *e. g.*, infantile eclampsia. From such a cause may arise a sudden stoppage in the development of the dental tissue, which, after a time, resumes its natural course, but with the result that the furrows or notchings remain as evidences of the recent condition.—*Archives of Pediatrics.*

## Figures and Tables

**Fig. 1 f1:**
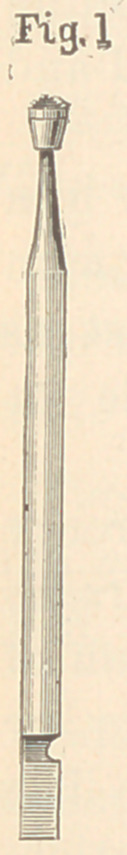


**Fig. 2 f2:**